# Experimental and spontaneous metastasis assays can result in divergence in clonal architecture

**DOI:** 10.1038/s42003-023-05167-5

**Published:** 2023-08-07

**Authors:** Antonin Serrano, Tom Weber, Jean Berthelet, Farrah El-Saafin, Sreeja Gadipally, Emmanuelle Charafe-Jauffret, Christophe Ginestier, John M. Mariadason, Samantha R. Oakes, Kara Britt, Shalin H. Naik, Delphine Merino

**Affiliations:** 1 Olivia Newton-John Cancer Research Institute, Heidelberg, VIC 3084 Australia; 2 School of Cancer Medicine, La Trobe University, Bundoora, VIC 3086 Australia; 3 Immunology Division, The Walter and Eliza Hall Institute of Medical Research, Parkville, VIC 3052 Australia; 4 Department of Medical Biology, The Faculty of Medicine, Dentistry and Health Science, The University of Melbourne, Parkville, VIC 3010 Australia; 5 CRCM, Inserm, CNRS, Institut Paoli-Calmettes, Aix-Marseille University, Epithelial Stem Cells and Cancer Laboratory, Equipe labellisée LIGUE contre le cancer, Marseille, 13009 France; 6 Garvan Institute of Medical Research, Darlinghurst, NSW 2010 Australia; 7 St Vincent’s Clinical School, UNSW Sydney, Darlinghurst, NSW 2010 Australia; 8 Breast Cancer Risk and Prevention Lab, Peter MacCallum Cancer Centre, Melbourne, VIC 3000 Australia; 9 Sir Peter MacCallum Department of Oncology, The University of Melbourne, Melbourne, VIC 3000 Australia

**Keywords:** Tumour heterogeneity, Cancer models

## Abstract

Intratumoural heterogeneity is associated with poor outcomes in breast cancer. To understand how malignant clones survive and grow in metastatic niches, in vivo models using cell lines and patient-derived xenografts (PDX) have become the gold standard. Injections of cancer cells in orthotopic sites (spontaneous metastasis assays) or into the vasculature (experimental metastasis assays) have been used interchangeably to study the metastatic cascade from early events or post-intravasation, respectively. However, less is known about how these different routes of injection impact heterogeneity. Herein we directly compared the clonality of spontaneous and experimental metastatic assays using the human cell line MDA-MB-231 and a PDX model. Genetic barcoding was used to study the fitness of the subclones in primary and metastatic sites. Using spontaneous assays, we found that intraductal injections resulted in less diverse tumours compared to other routes of injections. Using experimental metastasis assays via tail vein injection of barcoded MDA-MB-231 cells, we also observed an asymmetry in metastatic heterogeneity between lung and liver that was not observed using spontaneous metastasis assays. These results demonstrate that these assays can result in divergent clonal outputs in terms of metastatic heterogeneity and provide a better understanding of the biases inherent to each technique.

## Abstract (web summary)

Static cell barcoding shows spontaneous and experimental metastasis assays led to different clonal outputs, demonstrating the importance in considering experimental approaches for breast cancer modelling.

## Introduction

Despite improvements in the standard of care, breast cancer is still a leading cause of cancer-related death in women^1^. Among the breast cancer subtypes, triple-negative breast cancer (TNBC) lacks targeted therapy options and remains the deadliest subtype. The mortality rate of TNBC is linked with the distant spread of the disease that forms lethal metastases in vital organs^2^. Despite the dire need to better understand the advanced setting of the disease, study of the metastatic cascade is challenging^3^. Cancer cell fitness can be investigated in 2D or 3D cultures, but these models often lack the complex interaction with cells from the tumour microenvironment^4^. On the other hand, in vivo models offer the opportunity to study tumour progression within the tumour microenvironment and allow examination of the biology of metastases within their niche.

Cancer research has relied on the use of animal models for decades^5^. Historically, two models of metastases have been used side by side to investigate cancer progression. In the spontaneous metastasis assay, tumour cells are injected at an orthotopic or ectopic site to form a primary tumour. Metastases subsequently emerge through the spreading of tumour cells into the vasculature and survival in distant sites. In the experimental metastasis assay, tumour growth in the primary site is bypassed, and cells are delivered directly into the circulation for direct seeding of distant organs.

In spontaneous metastasis assays, the establishment of primary tumours can be challenging, which has led to the development of distinct transplantation methods^6^. Breast cancer xenografts or allografts were initially noted to be poorly metastatic^7^. However, cancer cells seemed to be more metastatic when injected orthotopically than when injected subcutaneously^8^. This underscores the importance of the tumour microenvironment during metastasis. For spontaneous breast cancer models, mammary fat pad transplantations can be performed in two ways: the endogenous mouse epithelium is ‘cleared’ and tumour cells are injected in the fat, or the epithelium remains intact and cancer cells are directly injected into the mammary gland^9^. More recently, intraductal injections have been developed to promote the establishment of ductal carcinoma in situ and luminal breast tumours within the duct^10,11^, where these tumours originate. This assay allows a better engraftment of oestrogen-positive tumours despite slower tumour growth compared to fat pad injections^12^. In TNBC, intraductal syngeneic models demonstrated a slower metastasis rate than mammary fat pad injection models^13,14^. Overall, intraductal injection is an appealing strategy to preserve the mammary gland and mimic the physiological process associated with tumour development, as cancer cells must pass through the basement membrane of the mammary duct to expand and metastasise.

In experimental metastasis assays, cells are most often injected directly into the tail vein, and they are known to be trapped in lung capillaries several minutes after the injection^15–17^. Other experimental modes of injection are commonly used to transplant cells into specific organs, such as intrasplenic injection (for development of liver metastases)^18^, intracardiac injection (for development of various metastatic sites, including brain and bone)^19,20^, intra-bone marrow injection^21^ and intracranial injection^22^.

While experimental metastasis models enable the investigation of the late stages of the disease in a time-effective manner^23^, they do not recapitulate the early steps of the metastatic cascade upstream of the intravasation of tumour cells into the systemic circulation. Spontaneous metastasis models can be used to study the metastatic process from the establishment of clones in the primary tumour. However, these assays may differ in the resulting cellular composition of the metastases they generate. Previous sequencing experiments suggest that lung nodules obtained from intravenous (IV) and intramammary fat pad (IMFP) transplantations have different gene expression profiles^24,25^, but these results have not been supported by other studies^24,26^.

To study the clonal dynamics responsible for metastatic growth in both assays, we labelled individual cancer cells using a lentiviral-based library of genetic barcodes^27^. As the DNA tags are stably integrated into the genome, they will be transmitted to daughter cells^28^. This strategy has been used previously in breast cancer research to interrogate clonal fate using cancer cell lines in vitro^29^ and patient and cell line xenografts in spontaneous^30–34^ or experimental in vivo assays^35,36^. Interestingly, serial IMFP transplantation experiments revealed that 1/10 to 1/10,000 cells transplanted into the mammary fat pad can survive and grow as clones (defined here as uniquely barcoded cells and their progenies), depending on the number of passages and models^30^. Indeed, heterogeneous tumours contain a diverse population of clones, presenting different levels of fitness and invasiveness after transplantation. Both spontaneous and experimental metastasis assays have shown that clonal fitness also varies significantly in metastatic niches, as the tumour microenvironment plays an important role in shaping metastasis heterogeneity^27,37,38^. Therefore, the site and method of injection in preclinical models might have crucial implications on clonal fate.

In this study, we compared the fate of genetically labelled human breast cancer clones in spontaneous and experimental metastasis assays using the MDA-MB-231 cell line and a TNBC PDX model. The barcode repertoire of primary and secondary lesions was analysed and demonstrated that these assays led to different clonal outputs in these models. This study provides compelling evidence to guide experimental design in breast cancer research, taking into consideration the heterogeneity of the disease.

## Results

### The site of injection has a minor impact on the growth kinetics of primary tumours, except for intraductal injections

**Fig. 1:**
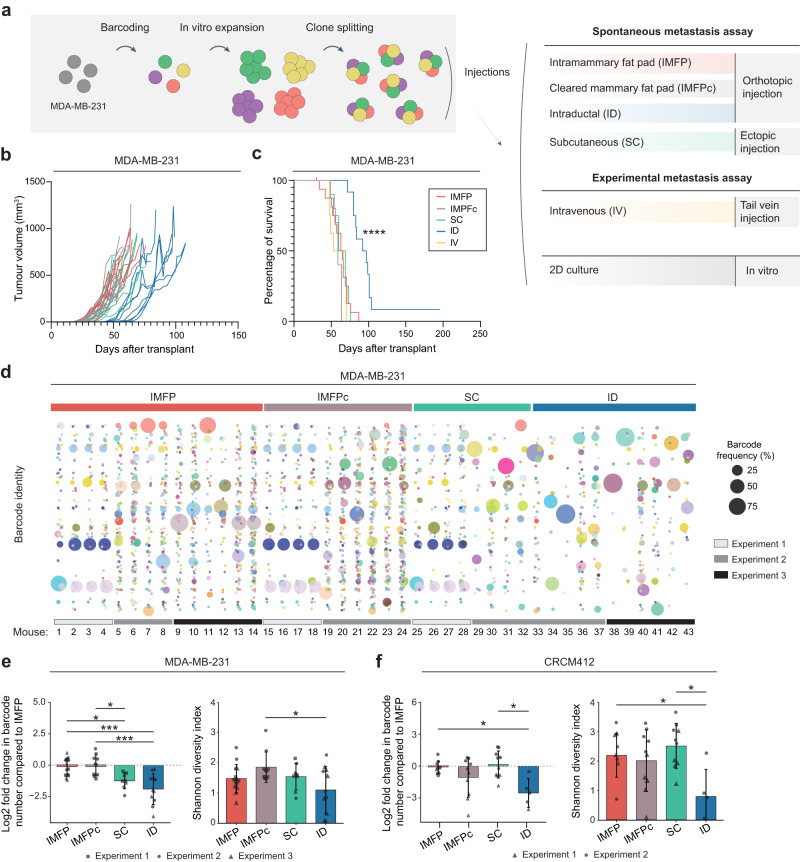
Kinetics of primary tumour growth after mammary fat pad, subcutaneous and intraductal transplantations of barcoded cells. **a** Cancer cells were genetically labelled before a short in vitro amplification and injection into recipient mice using different transplantation assays. Some cells were kept in vitro for the duration of the experiments. **b** Individual tumour growth curves for MDA-MB-231 and **c** Kaplan‒Meier survival plots, for different modes of injection. Intramammary fat pad injection (IMFP, *n* = 14, red), intramammary fat pad with clearing (IMFPc, *n* = 10, brown), subcutaneous injection (SC, *n* = 8, green), intraductal injection (ID, *n* = 11, blue), intravenous injection (IV, *n* = 8, yellow). Three independent experiments, log-rank (Mantel‒Cox) test, ****p value < 0.0001 for ID vs other injection modes survival. **d** Barcode composition of MDA-MB-231 tumours represented in a bubble plot. Each coloured dot represents a barcoded clone, and its size is correlated with its frequency. **e** Log2-fold change in barcode number, compared to IMFP (left panel) and Shannon diversity index (right panel) for MDA-MB-231 tumours. **f** Log2-fold change in barcode number, compared to IMFP (left panel) and Shannon diversity index (right panel) for PDX CRCM412 tumours. **e**, **f** Each point represents a tumour. Error bars represent the standard deviation of the means (SD) and the shape represents independent experiments. IMFP *n* = 14, IMFPc *n* = 10, SC *n* = 8, ID *n* = 11, 2–3 independent experiments for MDA-MB-231 and IMFP *n* = 10, IMFPc *n* = 10, SC *n* = 10, ID *n* = 5, 2 independent experiments for PDX CRCM412. Significance was determined by one-way ANOVA followed by Tukey multiple comparison test, *p value < 0.05, ***p value < 0.0005.

To investigate the impact of the mode of injection on intratumoural heterogeneity, a TNBC cell line, MDA-MB-231, and a treatment-naïve TNBC PDX, CRCM412, were barcoded with lentiviruses containing a library of 2,500 genetic barcodes, as previously described^32^. For the MDA-MB-231 cell line, two barcoded populations were generated. Both populations were first expanded in vitro prior to transplantation and injected into several mice via different routes to track whether their fate was consistent between mice (Fig. 1a). One population was used for Experiment 1, and the other population was used for Experiments 2 and 3. For PDX CRCM412, an in vitro expansion step was not possible, as the amplification of this PDX has not been adapted for in vitro culture. Therefore, for this model, a similar number of cells with different barcode repertoires were injected into several mice (Supplementary Fig. 1a).

The barcoded cells from the cell line and PDX were orthotopically injected via three different approaches: in the intact mammary fat pad (IMFP), in a mammary fat pad cleared from the endogenous epithelium (IMFPc), or intraductally (ID). Cells were also ectopically injected subcutaneously (SC). In parallel to these four spontaneous metastasis assays, cells were also injected in an experimental metastasis assay via intravenous (IV) tail vein injection (Fig. 1a and Supplementary Figure 1a). Of note, as described in the methods section, while the same number of cells were injected for each mode of injection within a given experiment, the volume of injection and the age of the mice varied depending on the assay.

Interestingly, using the MDA-MB-231 model, tumour growth was similar for mammary fat pad (if the fat pad was cleared or not cleared) and subcutaneous injection (Fig. 1b, c and Supplementary Fig. 1b, c). However, when injected in the mammary duct, tumours grew at a slower rate, in agreement with previous studies^12,13^. Clearing of the mammary gland was confirmed by staining of the glands post-surgery (Supplementary Fig. 1d). Intraductal injections were controlled by injections of diluted trypan blue to confirm the presence of the injected solution in ducts (Supplementary Fig. 1d). As expected, tumours injected in the fat pad had a more spherical appearance than tumours injected into the duct (Supplementary Fig. 1e).

Similar observations were obtained with the PDX CRCM412 (Supplementary Fig. 1f, g). While the same number of cells were injected in different conditions, tumour growth was found to be comparable between different modes of injection except for intraductal injections.

### The number of clones surviving in primary tumours varies according to the site of injection

Since the method of injection had a minor impact on the kinetics of primary tumour growth, their barcode composition was investigated next. When the primary tumours reached their ethical endpoint size (800 mm^3^), tumours were dissected into pieces and lysed to extract their DNA. This was followed by PCR amplification of the barcodes and next-generation sequencing.

Barcode analysis revealed that tumours resulting from mammary fat pad, subcutaneous and intraductal transplantations were all dominated by a handful of barcodes (~5–10 barcodes, Fig. 1d, Supplementary Figs. 1h, i and 2a), as previously described for IMFP transplantations^32,33,38^. When comparing the barcode repertoire in the MDA-MB-231 cohort, within and across different modes of injection, we found that primary tumours from different mice had a similar barcode composition in Experiment 1, where 200,000 cells were injected per mouse. However, this was not the case in the other 2 experiments, where only 60,000 cells were injected per mouse for each mode of injection, due to the limited injection volume required for ID experiments (Fig. 1d and Supplementary Fig. 2a). Surprisingly, tumours from experiment 1 overall contained fewer barcodes than tumours from experiments 2 and 3 (Fig. 1d and Supplementary Fig. 2a), despite a larger number of cells being injected. As these populations were generated independently, these differences in clonal engraftment could be due to differences in the genomic heterogeneity of the initial populations. When comparing the number of barcodes within each experiment, we found that IMFP and IMFPc transplantations contained the largest number of barcodes, followed by SC and ID injections (Fig. 1e).

In the PDX CRCM412 cohorts, barcode identity between tumours couldn’t be compared within and across modes of injection, as each mouse received a unique pool of barcoded cells (Supplementary Fig. 1a, i). However, the same number of cells were injected into multiple mice, enabling us to compare the percentage of engraftment in different conditions. We found that intraductal injections resulted in the lowest number of barcodes in primary tumours compared to the other groups (Fig. 1f). As a result, the Shannon diversity index, taking into consideration the number of barcodes and their frequency within the tumours^39^, was similar across all models, with the exception of tumours from the intraductal group, which were significantly less diverse in the PDX CRCM412 model (Fig. 1f).

One major difference between ID injections and all the other spontaneous metastasis assays was the absence of Matrigel in the injection buffer. Therefore, we investigated the impact of the presence or absence of Matrigel on tumour growth and clonal survival in IMFP and ID transplantations using the MDA-MB-231 model (Supplementary Fig. 2b, c). Interestingly, the absence of Matrigel slightly reduced the growth rate of cancer cells when injected in the mammary fat pad (Supplementary Fig. 2b) but not in the duct (Supplementary Fig. 2c). More importantly, in terms of intratumoural heterogeneity, the presence of Matrigel in the injected solution did not influence the barcode repertoire of the tumours from either IMFP or ID injections (Supplementary Fig. 2d). Therefore, the difference observed between IMFP and ID injections in terms of clonal growth is unlikely related to the presence of Matrigel.

As we observed differences between experimental conditions and between mice within a given condition in the MDA-MB-231 cohorts, we next analysed the hierarchical clustering of primary tumour samples based on Pearson correlations (Supplementary Fig. 3). As expected, samples from Experiment 1 clustered together and showed a low correlation with samples from Experiments 2 and 3, as they came from a different barcoded population. Within Experiment 1, the clonal repertoires of primary tumours were highly correlated, regardless of their mode of injection. Within Experiments 2 and 3, the same observation was made, but intraductal injections resulted in different clonal outputs compared to other modes of injection.

Taken together, these results demonstrated some similarities in intratumour heterogeneity in the MDA-MB-231 model, regardless of the mode of injection, the clearing of the mammary fat pad and the presence of Matrigel. However, intraductal injections significantly reduced clonal growth compared to the other modes of injection, likely due to the selective pressure of the tumour microenvironment.

### In vitro culture heterogeneity drift and relationship with tumours

We next compared the clonal composition of MDA-MB-231 barcoded cells grown as primary tumours in vivo versus in vitro. To do so, the same number of cells injected into mice (60,000 cells per mouse) were kept in culture in 4 flasks (60,000 cells per flask) at the start of experiments 2 and 3.

**Fig. 2:**
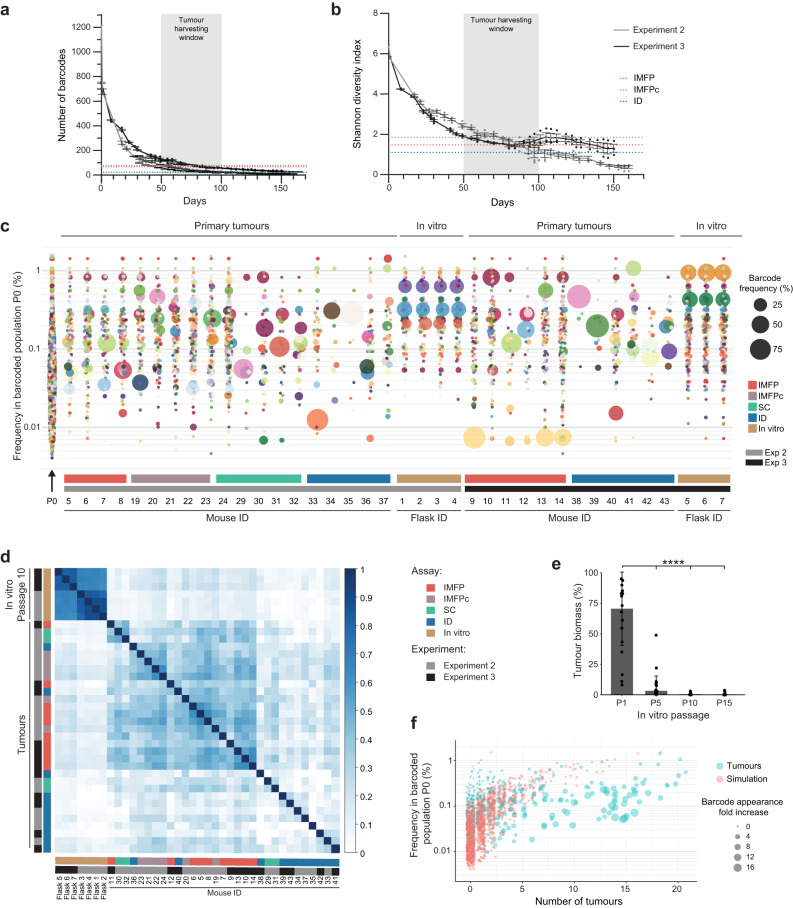
Study of clonal drift in the MDA-MB-231 cell line, in vitro and in vivo. **a** Total number of barcodes in each population, in culture, over the passaging time course. **b** Shannon diversity index of the in vitro population, over the passaging time course. The shadow area represents the time when primary tumours were harvested. **a**, **b** Coloured dashed lines represent the mean Shannon diversity index of IMFP (red), IMFPc (brown) and intraductal (blue) tumours. *n* = 4 for Experiment 2, *n* = 3–4 for Experiment 3, and for the initial populations, *n* = 1 for P0 and *n* = 2 for P1. Error bars represent the standard deviation of the means (SD). **c** Bubble plot representing the barcode composition of the P0 population (black arrow), primary tumours and in vitro cells (at passage 10, P10). The size of the dot is proportional to the frequency of the barcode. **d** Pearson correlation matrix of primary tumours and in vitro MDA-MB-231 barcoded cells from passage 10. **e** Median primary tumour biomass in percentage captured with top 95% barcodes present in in vitro flasks from passages 1, 5, 10 and 15. *n* = 16 tumours from 2 independent experiments. Significance was determined by one-way ANOVA followed by Tukey multiple comparison test, ***p value < 0.0005. Error bars represent the standard deviation of the means (SD). **f** Barcode detection in multiple tumours. Each dot represents a barcode. Barcodes are ranked according to their frequency at P0 on the *y*-axis, and the number of tumours containing these barcodes (*x*-axis). In red, simulation based on P0 frequency; in blue, empirical data. Dot size highlights the fold change increase in the number of appearances of the barcode in tumours (blue) compared to expected appearance (red).

Over a period of 4 to 5 months, cells were passaged twice per week, and at each passage, 10% of the population was sequenced to assess barcode number (Fig. 2a) and diversity (Fig. 2b). Strikingly, the number of barcodes and the Shannon diversity index of the populations in each flask were highly concordant between flasks at each passage (Fig. 2a–c). Moreover, a sharp decrease in barcode number and diversity was observed after the first passages (Fig. 2a, b). When the barcode number and diversity of the in vitro population (Fig. 2a, b, curves) were compared with those of the primary tumours grown in vivo at the time of tumour harvest (Fig. 2a, b, dashed lines), the overall heterogeneity was similar. However, while the results obtained in different flasks were highly correlated (Supplementary Figure 4a, b), a strong divergence in clonal identity was observed when comparing the results obtained in vitro (at passage 10, corresponding to Day 64 for Experiment 2 and Day 59 for Experiment 3) with the results obtained in vivo (corresponding to tumours harvested at Days 50–100; Fig. 2c, d). We then considered the dominant barcodes in vitro, contributing to 95% of the total frequency, at specific passages and analysed their median contribution within primary tumours. We found that the first passages in vitro recapitulated an important part of the primary tumour heterogeneity (Fig. 2e). However, this overlap was lost after five passages, to the extent that after 15 passages in vitro, the dominant barcodes in vitro only accounted for ~0.1% of the primary tumour biomass.

While clonal identity differed in vitro and in vivo (Fig. 2c–e), barcodes thriving in vivo tended to be present in multiple tumours with a certain degree of determinism (Fig. 2f). Indeed, barcodes at a medium frequency in the initial population were present in a greater number of tumours compared to simulated results based on their initial frequency, suggesting that these barcodes were likely to harbour deterministic phenotypes to become dominant in vivo.

Altogether, these results highlight a distinct clonal behaviour between in vitro and in vivo settings, as previously described^33,36,38^, and suggest that none of the tumours injected orthotopically or ectopically contained clones suited for expansion in 2D settings in the MDA-MB-231 model. Taken together, these results corroborated previous observations that clonal fitness is intrinsically programmed and largely depends on the tumour microenvironment^29,33,38^.

### Clonal frequency in metastases correlates with clonal frequency in primary tumours from spontaneous metastatic assays

As the tumour microenvironment plays an important role in clonal selection, we next compared the distribution of the barcodes in primary and distant sites in spontaneous metastasis assays. When tumours reached 800 mm^3^, the tumours, lungs, blood and liver were collected. Of note, the number of cancer cells detected in metastatic sites was higher in the MDA-MB-231 xenograft than in the PDX CRCM412 model (Supplementary Fig. 4c, d), and no liver metastases were detected in PDX CRCM412.

**Fig. 3:**
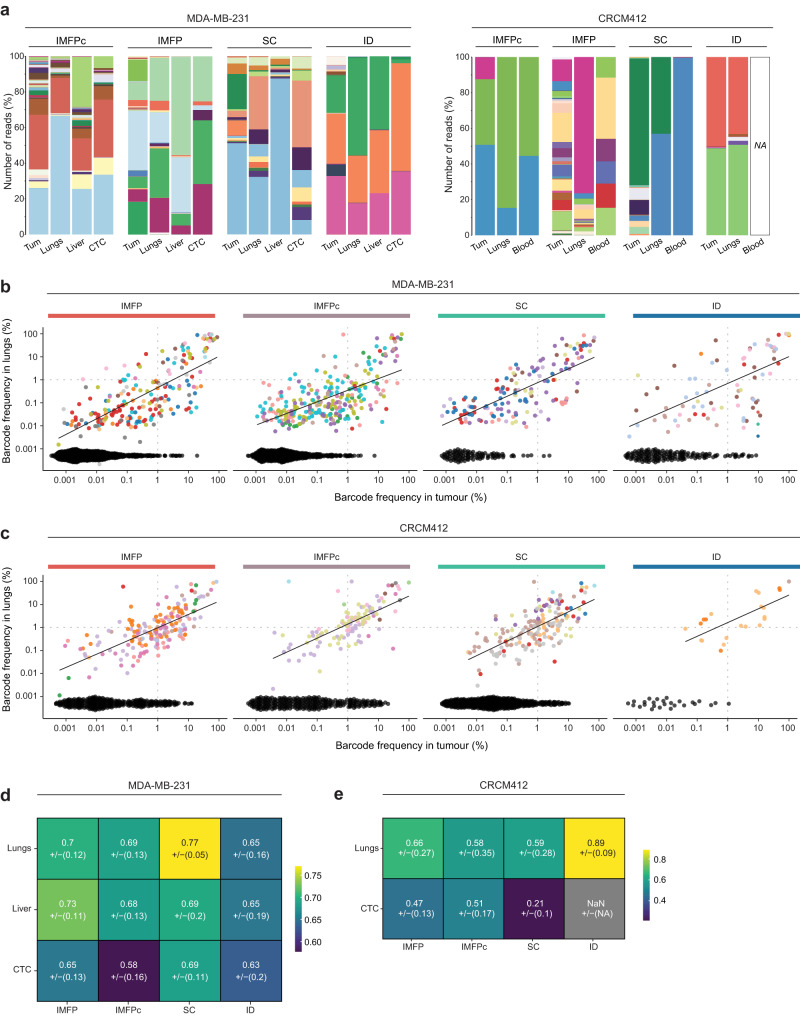
Comparison of barcode repertoire in primary and metastatic sites. **a** Example of the tumour, lung, liver, and CTC barcode composition as stacked histograms for each mode of injection using the MDA-MB-231 barcoded population (left panel) and PDX CRCM412 model (right panel). Each colour represents a barcode and its frequency is represented in the *y*-axis based on the percentage of reads. Scatter plots showing the clonal relationship between barcode frequency in primary tumours and lungs (in percentage) for each mode of injection for **b** MDA-MB-231 and **c** PDX CRCM412. Each dot represents a barcode. Black dots on the x-axis represent barcodes uniquely found in primary tumours. Dashed lines indicate a frequency of 1% in organs. Pearson values for the correlation between the barcode repertoire of tumours and distant sites for **d** MDA-MB-231 and **e** PDX CRCM412 models. SD are shown below the correlation values, between brackets. **b**, **d** For MDA-MB-231, *n* = 9 for IMFPc, *n* = 14 for IMFP, *n* = 8 for SC, and *n* = 11 for ID, over 2–3 experiments. **c**, **e** For PDX CRCM412, *n* = 10 for IMFPc, *n* = 10 for IMFP, *n* = 8 for SC and *n* = 3 for ID, over 2 independent experiments.

In both models, the frequency of barcodes detected in the blood and the lungs correlated positively with the frequency of barcodes in matching primary tumours in all spontaneous metastasis models (Fig. 3a–e and Supplementary Fig. 5a–c). This result indicated that the clones that were dominant in the primary tumour were more likely to be dominant in metastatic sites, regardless of the mode of injection. However, a few barcodes that were dominant in the lungs were only minor clones in primary tumours (Fig. 3b, c, top left quadrant) and vice versa (Fig. 3b, c, as indicated by the black dots on the x-axis). Similar observations were made in the liver of MDA-MB-231-bearing mice (Supplementary Fig. 5a) and blood from mice with MDA-MB-231 or PDX CRCM412 tumours (Supplementary Fig. 5b, c).

These results demonstrated that the high correlation of clonal frequency between primary and secondary sites previously described using IMPF transplantations^32,38^ was conserved in other spontaneous metastasis assays.

### The clonal repertoire detected in distant sites differs between spontaneous and experimental metastasis assays

As spontaneous assays (using IMFP injections, subcutaneous injections or intraductal injections) and experimental assays (using tail vein injections) are routinely used in metastasis research, we compared metastatic burden in both types of assays. As the metastatic niches are the same in both models and clonal fitness is largely dependent on the tumour microenvironment, we hypothesised that the same clones would have a similar growth advantage to form macro-metastases in distant organs. However, we anticipated that the direct injection of cells in the tail vein might favour the growth of clones that were not detected in spontaneous metastasis assays, for instance, clones that did not have the ability to intravasate.

In terms of metastasis burden, we found that the number of MDA-MB-231 cells detected in the lungs, blood and liver were similar in spontaneous and experimental assays (Supplementary Fig. 4c), with most of the mice reaching ethical endpoints at the same time after tumour cell injection (except for the intraductal group; Fig. 1b, c). However, strikingly, when 5000 barcoded cells from PDX CRCM412 were injected in the tail vein, no cells were observed in the lungs after 200 days (Supplementary Fig. 6a). However, the same number of barcoded cells transplanted in spontaneous assays formed a growing tumour able to shed cells detectable in the blood and lungs (Supplementary Figs. 4c and 6b). This difference could be explained by the low number of cells injected in these assays due to limitations in the size of the barcoding library. Indeed, the frequency of metastatic ‘seeders’^32^ in this model might be inferior to 1/5000. In this case, the injection of a larger number of cells would enable the initiation of lung metastases after tail vein injections. In contrast, MDA-MB-231 cells are a well-established cell line derived from a pleural effusion with a high seeding potential^38^. These cells colonised the lungs in both spontaneous and experimental metastatic assays with similar kinetics (Fig. 1c and Supplementary Fig. 4c, d).

**Fig. 4:**
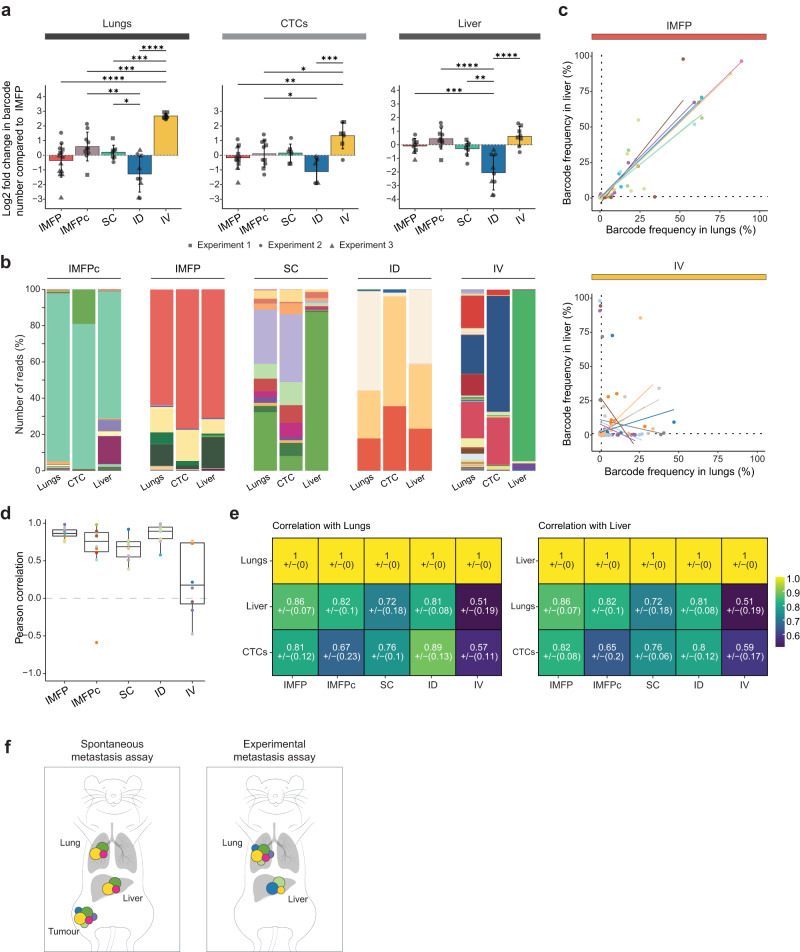
Comparison of metastatic burden and clonality in experimental and spontaneous metastasis assays in the MDA-MB-231 cell line. **a** Log2-fold change in barcode number, compared to IMFP (left panel) in lungs, liver and CTCs in mice injected with barcoded MDA-MB-231 for each mode of injection. Each shape represents an independent experiment. IMFP *n* = 14, IMFPc *n* = 10, SC *n* = 8, ID *n* = 9, IV *n* = 8, from 2 to 3 independent experiments for MDA-MB-231 One-way ANOVA followed by Tukey multiple comparison test, *p value < 0.05, **p value < 0.005, ***p value < 0.0005, ****p value < 0.00001. Error bars represent the standard deviation of the means (SD). **b** Examples of barcode composition in multiple organs represented as stacked histograms for each mode of injection using MDA-MB-231 barcoded cells. **c** Clonal relationship between lung and liver barcode repertoires represented as scatter plots for the MDA-MB-231 model injected in the mammary fat pad (top panel) and intravenously (bottom panel). Each dot represents a barcode, and each colour represents a mouse. Fitted lines represent individual animals (IMFP *n* = 13, IV *n* = 8, from 2 to 3 independent experiments). **d** Pearson correlation between the top 10 barcodes found in the lungs and liver. **e** Pearson correlation values between barcode repertoires in different sites for the MDA-MB-231 model, according to the injection mode. **f** Schematic overview of clonal spread in spontaneous and experimental metastasis assays in the MDA-MB-231 model.

As expected, the number of barcodes present in the lungs was significantly higher when MDA-MB-231 barcoded cells were injected in the tail vein compared to spontaneous metastasis assays, regardless of the mode of injection (Fig. 4a and Supplementary Fig. 6c). This result can be explained by the fact that cancer cells are trapped in pulmonary capillaries shortly after tail vein injections^40^, independent of their ability to intravasate. As a result, lung metastases from IV-injected cells were more heterogeneous than samples from all the other routes of injection (Supplementary Fig. 6d), and their barcode composition differed from those of the other groups (Supplementary Fig. 6e). In contrast, the inherent heterogeneity detected in the blood and liver was less impacted (Fig. 4a and Supplementary Fig. 6d). Of note, comparing different modes of injection from spontaneous metastasis assays also presented some discrepancies. In particular, the lungs and livers of mice from the intraductal injection group yielded fewer barcodes and a lower diversity compared to the IMFP and SC injection groups, despite presenting a similar number of cells in these organs (Fig. 4a and Supplementary Figs. 4c and 6d). Similar observations were made in PDX CRCM412 (Supplementary Fig. 6b).

We next compared the heterogeneity of lung metastases from experimental models to primary tumours from MFP transplantation models in the MDA-MB-231 cell line. We found that the number of barcodes was similar (Supplementary Fig. 6f), despite a difference in the Shannon diversity (Supplementary Fig. 6g). This suggests that these cells, derived from a patient breast cancer pleural effusion, may have a similar ability to survive in the lungs and the MFP. However, a stronger pressure of selection was applied when the cells were injected under the skin or within the duct (Supplementary Fig. 6f, g).

More strikingly and unexpectedly, discrepancies in barcode dominance and identity were observed in metastatic organs between spontaneous and experimental metastasis assays. Indeed, in contrast to spontaneous metastasis assays, barcode relationships between the lungs and liver demonstrated a clear absence of correlation in IV-injected mice (Fig. 4b–e and Supplementary Fig. 7). These results contrasted with those from the intramammary fat pad group, as demonstrated by the clonal relationship of these two organs in MDA-MB-231 (Fig. 4c, e and Supplementary Fig. 8a) in spontaneous metastasis assays. These results suggested that different clones were trapped in the lung and liver when the cells were injected directly into the tail vein, although they had a similar ability to grow in these niches after MFP transplantation. Furthermore, frequencies of barcodes detected in the blood were correlated with frequencies of barcodes detected in lung and liver in both assays (Fig. 4e). However many clones present in the vasculature in the IV-injected group were not detected in the liver (Supplementary Fig. 7). In PDX CRCM412, no barcodes were detected in the liver, but the barcodes detected in lungs and blood also correlated in frequency, at least for the mammary fat pad transplantation experiments (Supplementary Fig. 8b, c).

Altogether, these results highlight important differences between spontaneous and experimental metastasis assays in terms of metastatic heterogeneity in the MDA-MB-231 model (Fig. 4f). While mouse survival and metastatic burden were similar in these settings, the two metastatic assays clearly resulted in different clonal fitness in metastatic sites and therefore may not be used interchangeably to study metastasis clonality and resistance to therapy.

## Discussion

Tumour heterogeneity is a driver of treatment resistance and metastatic spread in breast cancer. Therefore, investigating the cellular and molecular bases of tumour heterogeneity using in vitro and in vivo assays is critical^41^. Several preclinical models have been developed to mimic the biological events that occur in the human body^42^. Spontaneous metastasis assays, including intramammary fat pad injections, subcutaneous injections and intraductal injections, allow primary tumours to form prior to metastatic disease. However, in such models, metastases can occur after a long latency. On the other hand, experimental models, where the cells are directly injected within the systemic circulation, are often used to bypass the first steps in the cascade of events leading to the rapid establishment of metastases. Considering the impact of injection methods on the clonality of primary tumours and metastases is essential to better inform experimental design in cancer research. These considerations are extremely timely due to the increasing use of emerging technologies to study clonal fate.

When comparing several spontaneous metastasis assays, we found that tumour growth was similar for subcutaneous and intramammary fat pad transplantations. Moreover, the clearing of the fat pad did not provide any significant advantage in terms of tumour growth for either the MDA-MB-231 cell line or PDX CRCM412. However, the results obtained using intraductal transplantation were accompanied by a delayed onset of tumourigenesis compared to the other transplantation models. These differences between modes of injection could not be explained by the presence or absence of Matrigel in the MDA-MB-231 model. However, as the mammary duct volume is limited, the density of cells in the injected volume was different, as well as the age of the animals. These two factors may contribute to the differences in tumour growth observed between intraductal injection and other modes of injection in these models. Since intraductal injections recapitulate breast cancer growth in a noninvasive state, further invasion steps might delay tumour growth^10^.

Surprisingly, the tumour growth rates between intramammary fat pad and subcutaneous injection were similar in PDX CRCM412, and a similar number of barcoded clones survived when injected in the MFP or subcutaneously, despite striking differences in the composition of the tumour microenvironment at the site of injection. However, this result is in contradiction with a previous report using other models^43^ and with results obtained in the MDA-MB-231 cell line. These differences are therefore likely to be model dependent, highlighting different compatibility requirements between cancer clones and cells from the tumour microenvironment. In addition, no differences were observed in the heterogeneity of tumours from intramammary fat pad injections with or without clearing, suggesting that the clearing of the endogenous epithelium does not impact tumour growth and heterogeneity in these models and therefore may not always be required to study tumour progression.

Clone splitting experiments, where a barcoded population is expanded prior to separation of daughter cells after expansion into multiple experimental splits thereby containing the same clones, permitted us to investigate the stochasticity of clonal fate in these settings^27^. Interestingly, after transplantation of equal pools of MDA-MB-231 barcoded cells into multiple mice, we found that the tumours had a similar barcode composition in the first experiment, as previously described^36,38,44^, but not in the second and third experiments. As the number of transplanted cells was higher in the first experiment than in the other two, the initial number of cells per clone at the time of transplantation might influence individual clonal growth. Indeed, cancer cells might be subject to environmental pressures that can remodel the clonal composition of the tumours, such as proximity to stromal cells or blood vessels^45,46^. When a larger number of cells per clone is injected (as in the first experiment), the random deposition of cells at the injection site might be less likely to affect clonality. Nevertheless, in Experiments 2 and 3, we did observe barcodes with low frequency in the initial population that were present across multiple tumours, and this repetitive contribution could not be explained simply by their frequency at the time of injection. Furthermore, these barcodes were not dominant in vitro. These results confirmed previous results suggesting that specific clones displayed a deterministic phenotype to survive in vivo^27^.

Our results in both models also indicate that the barcode repertoire detected in metastases derived from spontaneous models was highly correlated with the primary tumour, in agreement with previous findings^32^. In this assay, the site of transplantation did not influence the clonal correlation between the primary tumour and metastases. It would be interesting to study the metastatic potential of these cells after resection of the primary tumour. Indeed, some of the barcoded cells detected in lungs and blood might be ‘shedders’, whose clonal frequency often correlates between primary and secondary sites^32^, rather than ‘seeders’ that are able to generate macro-metastases. This hypothesis coincides with the modest number of cells detected in distant organs in the absence of resection and with the absence of metastases after injection of these cells directly into the bloodstream. On the other hand, the MDA-MB-231 cell line is derived from a patient malignant pleural effusion. In this case, most of the clones detected in the periphery are likely to have a seeding capacity, in agreement with a previous study^38^.

In contrast to the highly correlated clonal distribution in tumour and metastatic sites in spontaneous metastasis assays, different clones were dominant in the lungs and liver after IV injections. These discrepancies between organs suggest that metastatic heterogeneity clearly depends on the route of injection in this model. The differences might be due to differences in the kinetics of dissemination and the frequency of the clones entering the vasculature. Indeed, in spontaneous metastasis assays, primary tumours may shed cells from dominant clones over weeks of tumour growth via the circulatory or lymphatic system, while in experimental metastasis assays, a more evenly distributed population of clones is forced into the blood flow at a given time. In the latter case, a proportion of cells might be trapped in the lungs, while another fraction extravasates in the liver as the first site. Therefore, lung and liver metastases might progress in parallel with distinct clonal repertoires. It would be interesting to see if this divergence is observed when injecting a larger number of cells per clone, increasing the probability of cells from each clone to reach both sites. These considerations have important implications in the study of metastatic heterogeneity using experimental models to bypass the tumourigenesis step.

In summary, this study provides meaningful information on the heterogeneity of tumours and metastases according to in vitro or in vivo assays, highlighting the similarities and differences of assays in the MDA-MB-231 model, which is routinely used in cancer research. These results also raise questions regarding why some clones can intravasate, extravasate and thrive in specific tumour microenvironments and what anatomical and molecular mechanisms fuel clonal selection and dynamics. Further investigations will be required to answer these questions, extend this study to other models and determine the nature of the processes dictating clonal fate in metastatic sites.

## Materials and methods

### Generation of barcoded MDA-MB-231 and PDX CRCM412 populations

The MDA-MB-231 cell line was obtained from ATCC at passage 39 (#HTB-26). Cells were maintained in RPMI medium (with Hepes, Thermo Fisher #22400036) supplemented with 10,000 U/ml penicillin/streptomycin and 10% foetal bovine serum (FBS). Cells were transduced for 48 h with lentivirus containing 2500 unique DNA barcodes^32,47^. Successfully labelled cells were isolated via flow cytometry for the expression of GFP. Infection efficiency in cell generation was kept low to ensure the integration of a unique barcode per cell. The GFP positivity was 7.1% and 5% for the first and second populations, respectively. Then, for each population, 25,000 barcoded cells were expanded in vitro for 7 days before another sorting to ensure that all cells in the population had been barcoded. Cells were then amplified and frozen. The first population was used for Experiment 1, and the second population was used for Experiments 2 and 3. Prior to injection, aliquots of the barcoded population were thawed and expanded for 7 days in culture before injection into recipient mice as described below. For in vitro experiments (Experiments 2 and 3), an identical number of cells to that used in the in vivo injection were independently plated in tissue culture flasks and maintained in culture for thirty passages. These independent cultures of cells were passaged prior to reaching confluency and kept in culture. Passaged cells were lysed, and the barcodes were amplified and sequenced to assess barcode heterogeneity, as described below.

The PDX CRCM412 model (Cancer Research Centre of Marseille) was generated at the Institut Paoli-Calmettes (France) from a drug-naïve TNBC patient tumour. To do so, 200,000 cells from an early passage tumour (passage 5) were orthotopically injected into the mammary fat pad of female NSG mice to be amplified prior to barcoding. Once harvested, tumours were processed as single-cell suspensions as previously described^38^. Cells were plated in 24-well plates (flat bottom ultralow attachment, #734-1584, Corning) at a density of 300,000 cells in 300 µl of mammosphere media composed of DMEM-F12 (#10565042, ThermoFisher) supplemented with 1X B27 (#17504001 ThermoFisher), 100 U/ml penicillin‒streptomycin (#15140122, ThermoFisher), 5 µg/ml insulin (#11376497001, Sigma‒Aldrich), 1 µg/ml hydrocortisone (#H0396-100MG, Sigma‒Aldrich), 0.8 U/ml heparin (#H0878-100KcU, Sigma‒Aldrich), 20 ng/mlbasic fibroblast growth factor (#01-106, Merck-Millipore) and 20 ng/ml epidermal growth factor (#E9644, Sigma‒Aldrich). Cells were infected with lentiviruses containing the barcode library as previously described^32^ at low MOI (Experiment 1: 9.1%, Experiment 2: 8.2%) to ensure the integration of a single barcode per cell. Barcoded cells were sorted for GFP positivity and resuspended in injection buffer as described below.

### In vivo assays

In vivo experiments were conducted in female immunodeficient NOD-scid gamma (NSG) mice. All procedures in animals were conducted in accordance with the Australian National Health and Medical Research Council guidelines under the approval of the Austin Animal Ethics Committee. The use of patient samples was approved by the Austin Health Human Research Ethics Committee.

The MDA-MB-231 barcoded population used for the in vivo experiments originated from the barcoded population previously established and frozen. For subsequent experiments, aliquots of the population were thawed and expanded for one week and then injected into recipient mice. After the initial thaw, 500,000 cells were sequenced to ensure similarity of the barcode repertoire (Supplementary Fig. 4a, b “control”). Interestingly, the barcode repertoire after the freeze and thaw cycle was still highly correlated with the initial P0 population, indicating no effect on clonal composition induced by thawing and quick expansion.

During the first experiment, 200,000 MDA-MB-231 cells were injected after preamplification of the infected population for each mouse and for each mode of injection. Only 60,000 cells were injected for Experiments 2 and 3 for each mouse and each mode of injection due to the limitation incurred by the cell density allowed for intraductal injection. However, 60,000 cells allowed the least frequent barcodes in the population to be represented based on initial population analysis.

For the PDX CRCM412 barcoded cells, 5000 cells were injected per mouse following the barcoding step. The frequency of tumour-initiating cells for this PDX is low (1-2%), thus minimising the risk of multiple repeats of similar barcodes in the injected population.

For intramammary fat pad injections (with and without clearing) and subcutaneous injections, cells were resuspended in injection buffer (42.5% DPBS, 30% FBS 25% Matrigel and 2.5% trypan blue) at the appropriate concentration to inject 5k or 60k cells for the PDX or MDA-MB-231 models, respectively, in a volume of 10 µl per injection. For injection controls without Matrigel, the volume of Matrigel was replaced by DPBS, and injections were performed similarly. Cells for lateral tail vain injection were resuspended in the appropriate volume to inject a similar number of cells in 100 µl of DPBS. Finally, cells for intraductal injections were resuspended in injection buffer without Matrigel (56.7% DPBS, 40% FBS and 3.3% trypan blue) for a total volume per injection of 4 µl.

Intramammary fat pad injections were performed in the fourth mammary glands of NSG mice (4- to 6-week-old animals). Intramammary fat pad injection with clearing was performed in 3- to 4-week-old female NSG mice, as previously described^48^. The region between the nipple and proximal lymph node was excised to remove the endogenous epithelium, and the tumour cells were injected into the remaining fat pad.

Intravenous injections were performed via the lateral tail vein of 4- to 6-week-old NSG females, and animals were warmed under a heat lamp 10 min prior to injection.

Intraductal injection was performed in 10- to 13-week-old female NSG mice with a 30 G blunt Hamilton syringe. The fur around the nipple area was removed with hair-removing cream, and the nipple was cut using spring scissors while holding the nipple. After cutting the nipple tip, a needle was inserted at a similar angle to the duct while firmly holding the base with tweezers. Once the needle tip was in the duct, 4 µl of injection buffer was slowly injected. After injection, one animal was sacrificed to evaluate injection into the ducts (Supplementary Fig. 1d).

### Tumour and organ processing

Tumours were harvested once they reached 800 mm^3^ and dissected into equal pieces. Blades were changed between each tumour to avoid barcode cross-contamination. Pieces of tumour were resuspended in 300 µl of lysis buffer (Viagen Biotech supplement with 1:50 proteinase K 20 mg/ml (Invitrogen)) and lysed overnight on a heater shaker set to 800 rpm at 55 °C, followed by 30 min at 85 °C and 5 min at 95 °C.

Blood was collected via cardiac puncture at the terminal endpoint prior to red blood cell lysis for 5 min at room temperature, followed by centrifugation at 500×*g* for 5 min. Pellets were resuspended in freezing media for PDX cells (for subsequent sort isolation). MDA-MB-231 cells were resuspended in 50 µl of PBS to be counted and then lysed in 50 µl of lysis buffer for 1 h at 55 °C, followed by 30 min at 85 °C, and finally 5 min at 95 °C on a heater shaker set to 800 rpm. Samples were stored in a freezer until PCR amplification.

Lungs from PDX CRCM412 and MDA-MB-231 models were collected at the ethical endpoint and viably frozen for subsequent single-cell sorting. Livers from the MDA-MB-231 model were also harvested. Tissues were manually chopped and digested in 5 ml of RPMI 1640 for MDA-MB-231 cells and in DMEM F12 (Thermo Fisher) for PDX cells. Both media were supplemented with 300 U/ml collagenase IA (Sigma Aldrich) and 100 U/ml hyaluronidase (Sigma Aldrich). Samples were incubated at 37 °C on an orbital shaker set at 300 rpm for 45 min and resuspended through an 18 G needle after 20 min and a 21 G needle after 40 min of digestion. The cell suspension was filtered through a 70 µm cell strainer and spun down for 5 min at 500×*g*. PDX samples were resuspended in DPBS with PI to be sorted via flow cytometry with BD FACSAria III cell sorter operating with BD FACSDiva software. Sorted cells were spun down and resuspended in 50 µl lysis buffer. MDA-MB-231 lung and liver single-cell suspensions were resuspended in 100 µl DPBS for counting and then resuspended in 100 µl lysis buffer. Lung and liver samples were lysed for 1 h at 55 °C followed by 30 min at 85 °C, and finally 5 min at 95 °C on a heater shaker set to 800 rpm. Samples were stored in the freezer until PCR amplification.

### In vitro expansion

MDA-MB-231 barcoded cells were plated at the same time as in vivo transplantation with similar numbers of cells in independent cultures (200,000 cells for Experiment 1, 60,000 cells for Experiments 2 and 3). Cells were maintained in culture media as previously described and passaged 1:10 prior to reaching confluency (twice weekly), and 1 ml of passaged cells per culture were pelleted and lysed in 100 µl lysis buffer for 1 h at 55 °C, followed by 30 min at 85 °C and finally 5 min at 95 °C on a heater shaker set to 800 rpm. Samples were stored at −20 °C until PCR amplification.

### Barcode amplification and sequencing

PCR amplification was performed on crude lysates. Tumour piece lysates were diluted 1:10 in water, and 40 µl of template was mixed with 160 µl of PCR mix in the well of a 96-well plate (#3420-00 S, SSIbio). They were then split into two technical replicates of 100 µl before the start of PCR to assess barcode detection reliability. Blood and lung samples were run in quintuplicate for PDX samples.

The first PCR run included the common primers (TopLib 5′-TGCTGCCGTCAACTAGAACA-3′ and BotLib 5′-GATCTCGAATCAGGCGCTTA-3′) to allow for barcode amplification. The cycle specifications were 94 °C for 5 min, followed by 30 cycles at 94 °C for 15 sec, 57.2 °C for 15 sec, 72 °C for 15 sec, and then 72 °C for 10 min. Products of the first PCR were then used to run the second PCR to add specific individual indices for NexGen sequencing (Supplementary Data 1). The cycle specifications of the thermocycler were 94 °C for 5 min, followed by 30 cycles at 94 °C for 5 sec, 57.2 °C for 5 sec, 72 °C for 5 sec, and then 72 °C for 10 min. All PCRs were run on an S100 thermal cycler (Bio-Rad), and the final PCR product at 266 bp was verified by 2.5% agarose gel electrophoresis. Samples were pooled and cleaned up with magnetic beads (#744100.4, Macherey-Nagel) before sequencing on Next-Seq (Illumina).

### Bioinformatic analysis of the barcode repertoire

Sequencing results were analysed on RStudio (Version 1.4.1106). Demultiplexing of sequencing FastQ files was performed using the ProcessAmplicon function from the edgeR package to generate a read-count matrix for each barcode per sample. To ensure the quality of the data, multiple filters were applied to the dataset. Once separated by sample, barcode read counts below or equal to 10 were set to zero and then replicated, and quintuplicate samples were used to filter out barcodes, *i.e*., barcodes present in fewer than 2 replicates were discarded. Replicates were then pooled by adding read count values of barcodes and normalised. Tumour pieces were processed similarly to re-create full tumours, and each barcode value for individual pieces was added and normalised as ‘Tumour sample’. In the MDA-MB-231 barcoded population used in Experiment 1, 3 barcodes (ID# 915, 1312, 1864) were identified at a similar frequency ratio in multiple samples, likely resulting from multiple integrations. Therefore, these barcodes were collapsed into a single virtual barcode. Figures were generated with the ggplot2 package.

### Statistics and reproducibility

Statistical analyses were performed with GraphPad Prism version 9.2.0. Unpair t-tests were used when comparing two groups. Comparisons between more than two groups were assessed using one-way ANOVA followed by Tukey multiple comparison tests. For Kaplan‒Meier survival curves, we used the log-rank (Mantel‒Cox) tests. All data in the bar graphs represent the means ± standard deviations (SDs) and *p* values < 0.05 were considered statistically significant.

### Reporting summary

Further information on research design is available in the Nature Portfolio Reporting Summary linked to this article.

### Supplementary information

Supplementary Information

Description of Additional Supplementary Files

Supplementary Data 1

Supplementary Data 2

Reporting Summary

## Data Availability

The data that support the findings of this study are available here: https://zenodo.org/record/8146196. Primer sequences for NexGen sequencing can be found in Supplementary Data 1. Numerical data supporting each graph included in main and supplemental figures can be found in Supplementary Data 2. Any remaining information can be obtained from the corresponding author upon reasonable request.

## Code availability

The code used to analyse the data is available here: https://zenodo.org/record/8146196.
