# Supplementary material for: Experimental and spontaneous metastasis assays can result in divergence in clonal architecture
Source: Commun Biol. 2023 Aug 7;6:821. doi: 10.1038/s42003-023-05167-5 (PMC10406815; doi:10.1038/s42003-023-05167-5)
Supplement: Supplementary file 1 — Supplementary Information [file 42003_2023_5167_MOESM1_ESM.pdf]

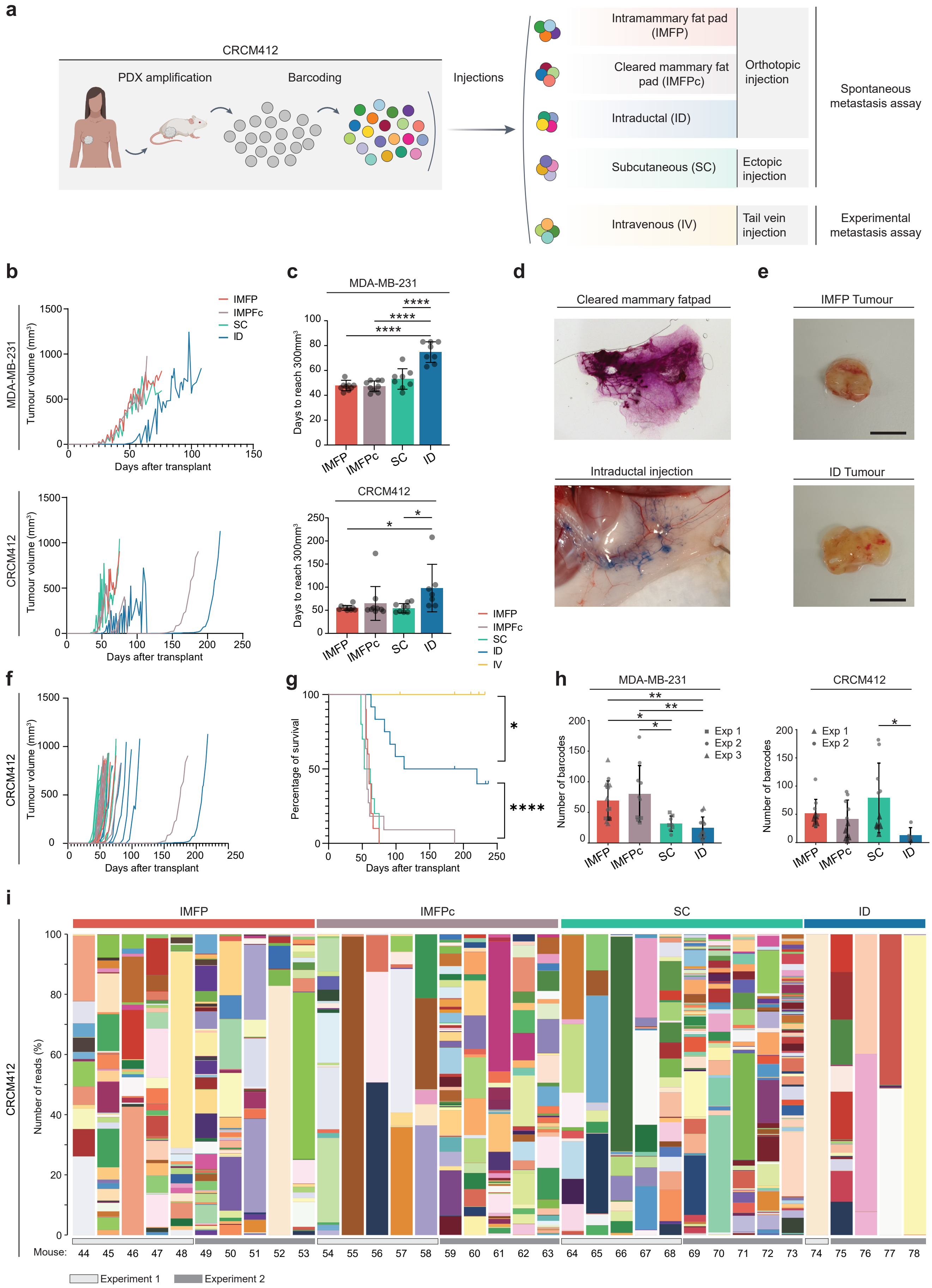

Supplementary Figure 1. a) Experimental design overview for PDX CRCM412. Cancer cells were genetically labelled before in vivo transplantation into recipient mice via different modes of injection. Some elements of the graphic were created with BioRender.com. **b)** Mean tumour volumes for each mode of injection for MDA-MB-231 (top panel) and PDX CRCM412 tumours (bottom panel). **c)** Number of days for MDA-MB-231 or PDX CRCM412 tumours to reach 300 mm3. IMFP n=14, IMFPc n=10, SC n=8, ID n=11, from 2 to 3 independent experiments for MDA-MB-231. IMFP n=10, IMFPc n=10, SC n=10, ID n=7, from 2 independent experiments for PDX CRCM412. **d)** Mammary fat pad collected post-clearing, mounted and stained with carmine alum (top), photo after intraductal injection to control presence in the duct (bottom). **e)** Photos of a dissected MDA-MB-231 primary tumour from a intramammary fat pad injection (IMFP, top) or an intraductal injection (ID bottom). Scale represents 10 mm. **f)** Individual growth curves in each mode of injection group for PDX CRCM412. **g)** Kaplan–Meier survival curves for PDX CRCM412, Log-rank (Mantel–Cox) test, *=p value < 0.05 for ID vs. IV, ****=p value < 0.0001 for ID vs. other injection modes. **h)** Number of barcodes detected in primary tumours from MDA-MB-231 or PDX CRCM412 models, the shape represents independent experiments. **i)** Barcode composition of CRCM412 tumours, represented as stacked histograms; each colour represents a barcode. Independent experiments are underlined in black (Experiment 1) or grey (Experiment 2). Intramammary fat pad injection (IMFP, n=10, red), intramammary fat pad with clearing (IMFPc, n=10, brown), subcutaneous injection (SC, n=10, green), intraductal injection (ID, n=12, blue), intravenous injection (IV, n=9, yellow) for CRCM412 tumours, over 2 independent experiments. **c-h**): One-way ANOVA followed by Tukey multiple comparison test, *=p value <0.05, **=p value <0.005, ***=p value <0.0005, ****=p value <0.0001. Error bars represent the standard deviation of the means (SD), with 2-3 independent experiments for MDA-MB-231 and 2 independent experiments for PDX CRCM412.

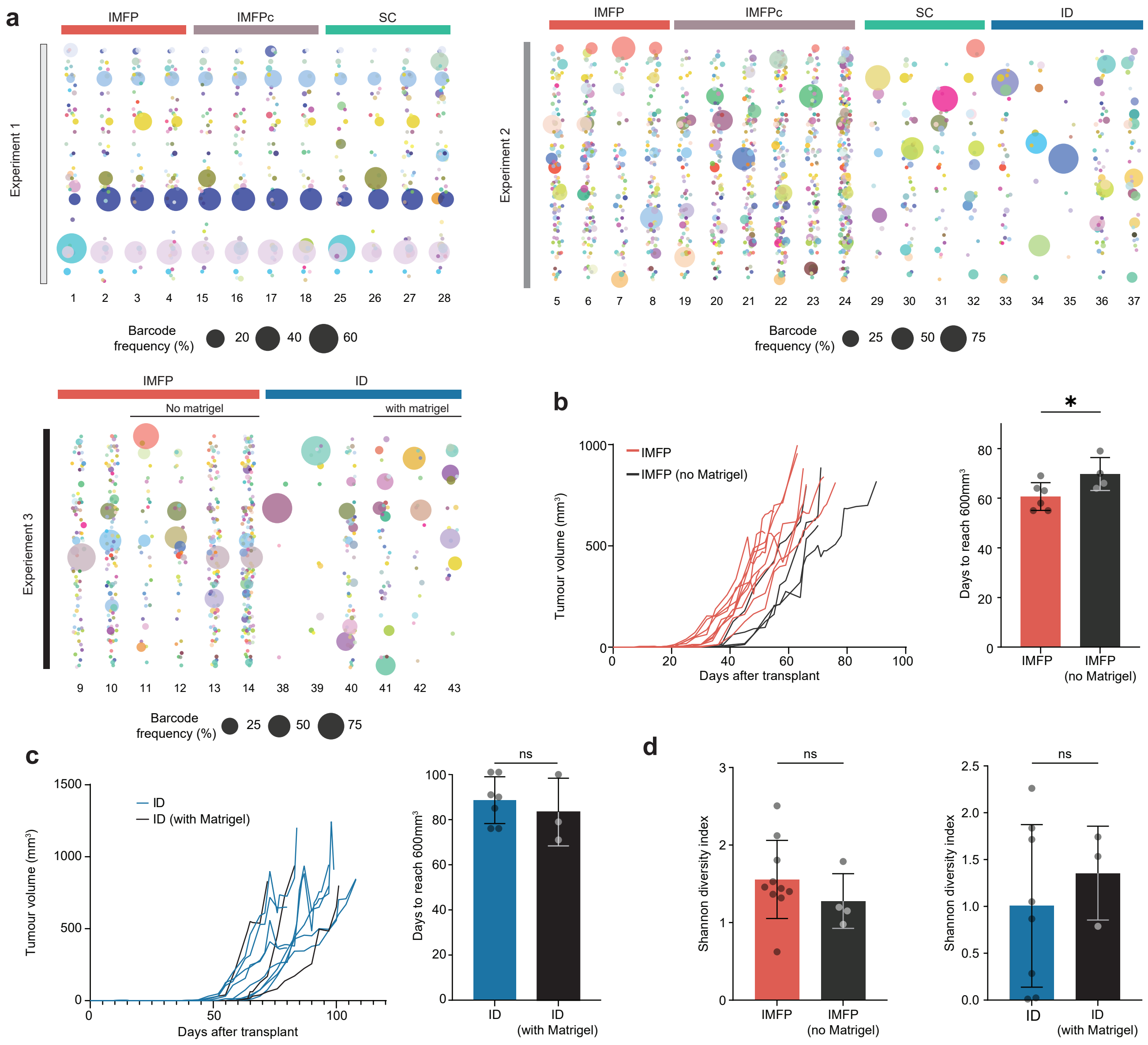

Supplementary Figure 2. a) Barcode composition of MDA-MB-231 barcoded primary tumours represented by bubble plots, separated by experiment. The colours of the barcodes are consistent with those used in Figure 1. Mouse numbers are indicated at the bottom of the graph. **b)** Growth of MDA-MB-231 tumours after intramammary fat pad injection of the barcoded cancer cells in the presence (red, n=6) or absence (dark grey, n=4) of Matrigel. The number of days for tumours to reach 600 mm3 is plotted on the side. IMFP n=6, IMFP (no Matrigel) n=4. **c)** Growth of MDA-MB-231 tumours after intraductal injection of the barcoded cancer cells in the presence (blue, n=7) or absence (black, n=3) of Matrigel. The number of days for tumours to reach 600 mm3 is plotted on the side. ID n=7, ID (with Matrigel) n=3. **d)** Shannon diversity index of tumours after intramammary fat pad and intraductal injections with and without Matrigel. IMFP n=10, IMFP (no Matrigel) n=4, ID n=8, ID (with Matrigel) n=3. **b-d)** Unpaired t-test, ns (non significant)=p value>0.05, *=p value <0.05, error bars represent the standard deviation of the means (SD).

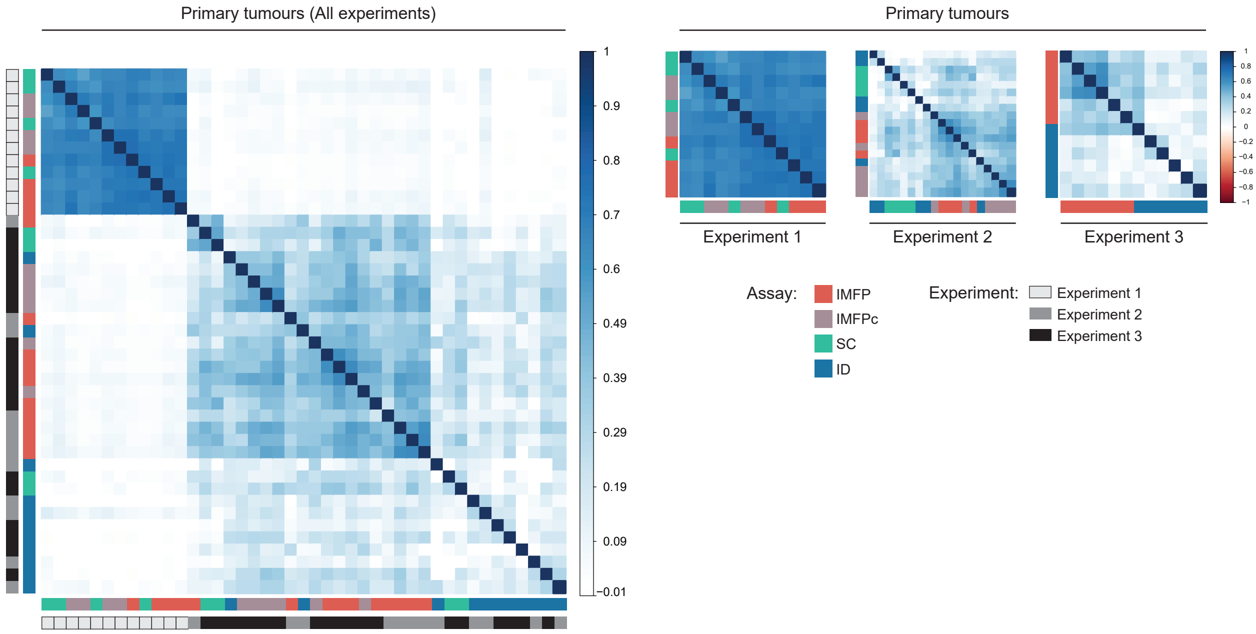

Supplementary Figure 3. Hierarchical clustering of Pearson correlations between MDA-MB-231 primary tumours from all experiments (left) and for each individual experiment (right).

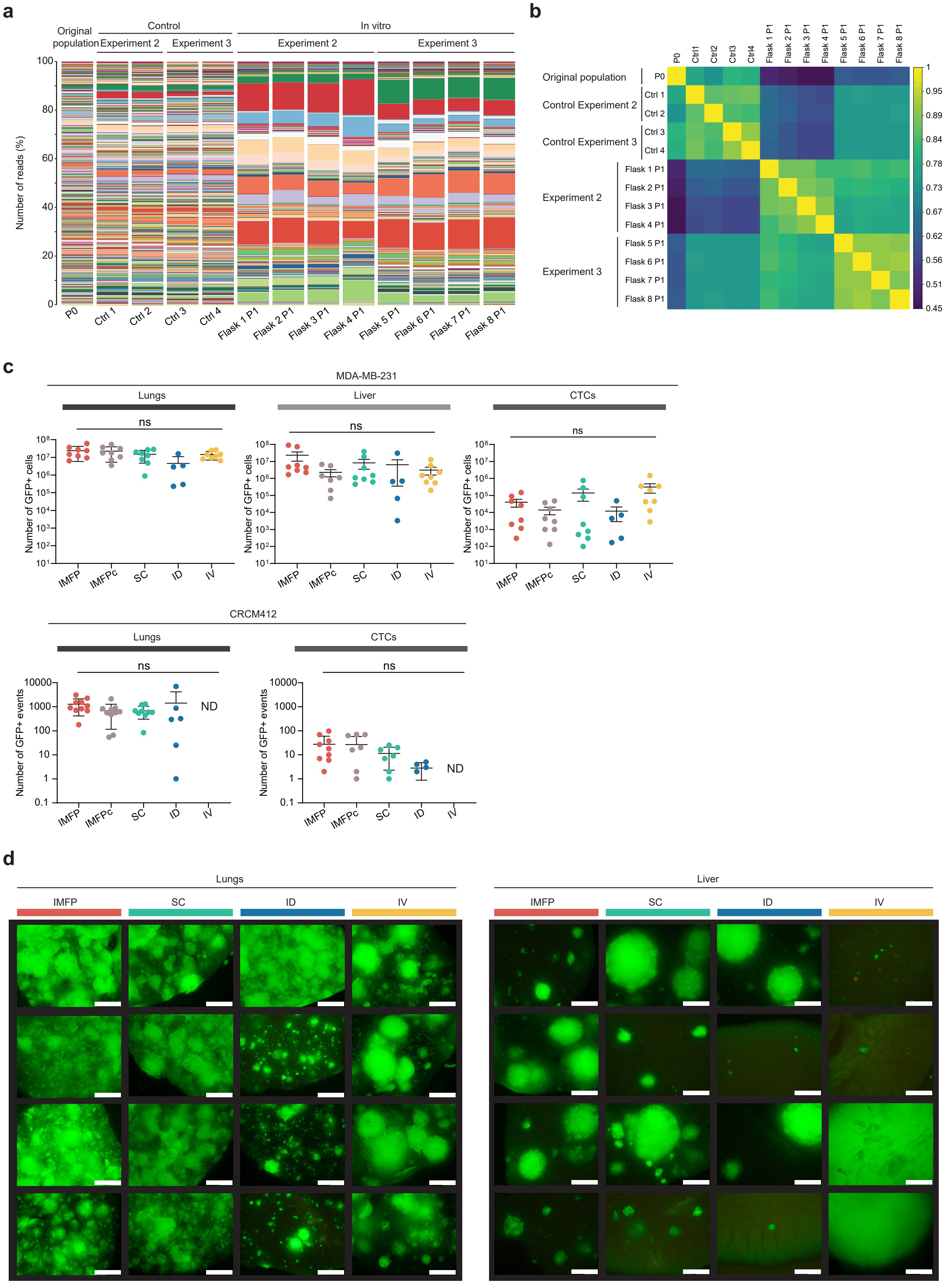

Supplementary Figure 4. a) Stacked histogram representing the barcode repertoire of the MDA-MB-231 barcoded populations: original population (P0), controls at the time of injection and in vitro seeding (Control) and individual flasks at the first passage (in vitro expansion) in 2 independent experiments (from the same original population). **b)** Pearson correlation corresponding to Panel a. **c)** Total number of cells found in lungs, liver and blood in the MDA-MB-231 model for each mode of injection at ethical endpoint (top row) and number of GFP+ cells detected in lungs and blood in the CRCM412 model. The ethical endpoint for spontaneous models (IMFPc, IMFP, SC, ID) refers to the primary tumour size reaching 800 mm3, while for the experimental model (IV), the ethical endpoint was reached when the mice got sick from metastatic disease. IMFP n=8, IMFPc n=8, SC n=8, ID n=5, IV n=8, from 2 to 3 independent experiments for MDA-MB-231. IMFP n=10, IMFPc n=10, SC n=9, ID n=6, IV n=4, from 2 independent experiments for PDX CRCM412. One-way ANOVA followed by Tukey multiple comparison test, ns (non significant)=p value>0.05. Error bars represent the standard deviation of the means (SD). **d)** Images of lungs and liver at ethical endpoint prior to organ digestion for each mode of injection using the MDA-MB-231 cell line. Cancer cells are GFP positive. Scale represents 1 mm.

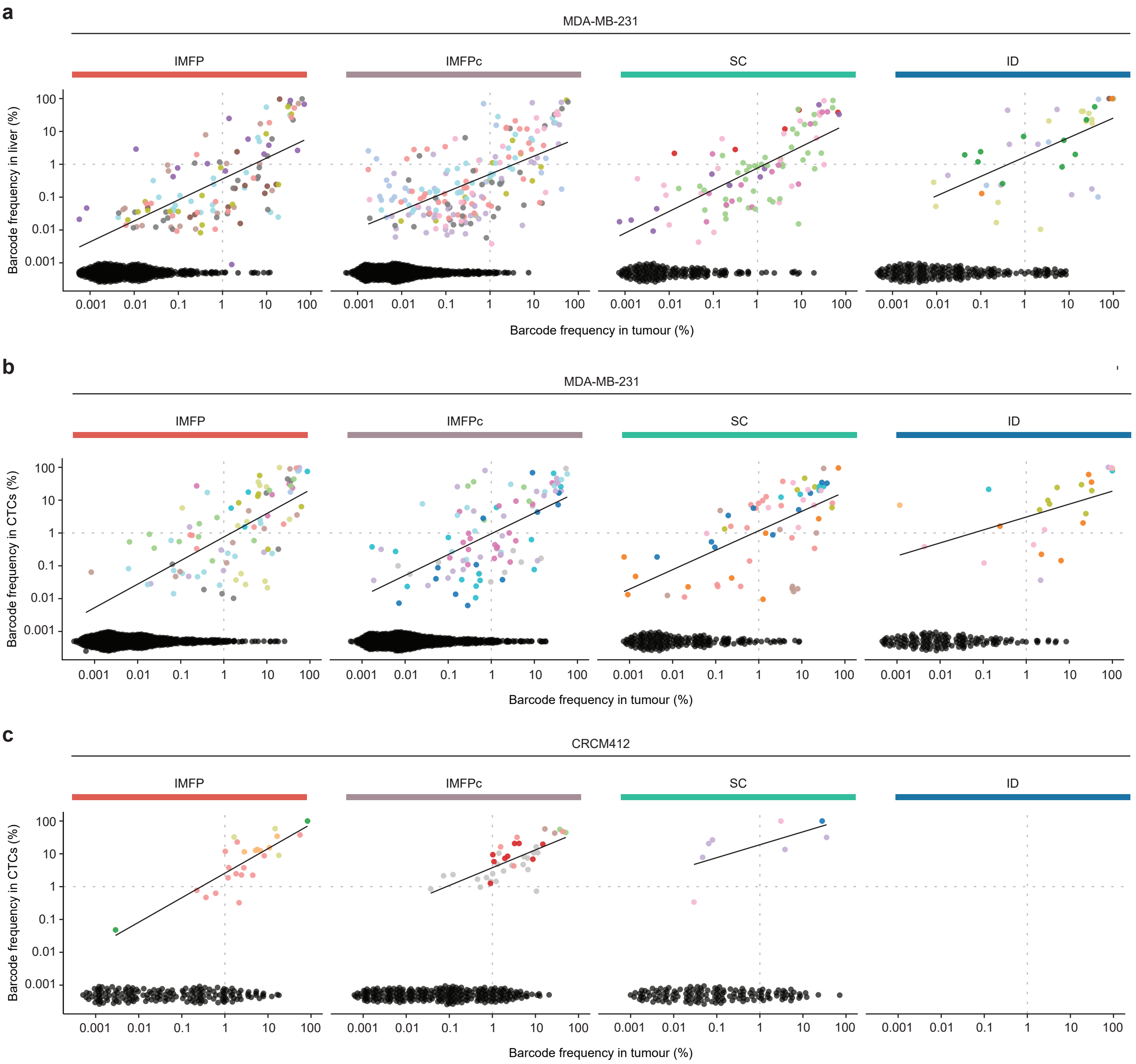

Supplementary Figure 5. a) Scatter plots representing barcode frequency in primary tumours and liver for MDA-MB-231 xenografts. Each dot is a barcode, and each mouse is represented by a colour. **b)** Scatter plots representing barcode frequency in primary tumours and CTCs for MDA-MB-231. **c)** Scatter plots representing barcode frequency in primary tumours and CTCs for PDX CRCM412. The dashed lines indicate 1% frequency. Black dot on the x-axis represents barcode not detected from y-axis.

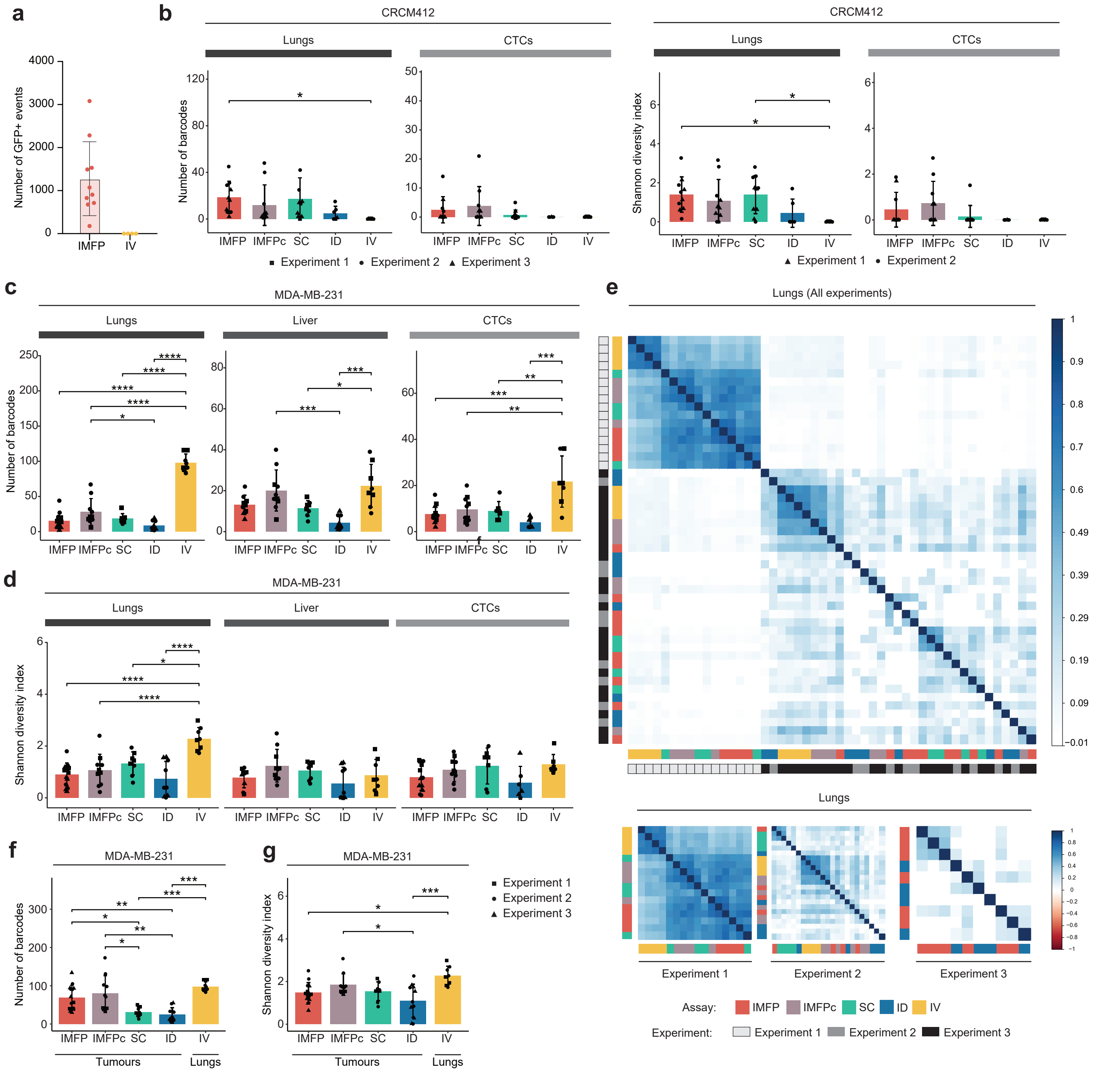

Supplementary Figure 6. a) Quantification of GFP-positive cells in the lungs, 200 days after intramammary fat pad or intravenous injections of PDX CRCM412 barcoded cells. IMFP n=10 and IV n=4. **b)** Total number of barcodes and Shannon diversity index for barcodes detected in lungs (left panel) and CTCs (right panel) in the PDX CRCM412 model for each mode of injection at ethical endpoint. The ethical endpoint for spontaneous models (IMFPc, IMFP, SC, ID) refers to the primary tumour size reaching 800 mm3, while for the experimental model (IV), the ethical endpoint was reached when mice got sick from metastatic disease. Each dot represents a mouse and the shape represents independent experiments. IMFP n=10, IMFPc n=10, SC n=9, ID n=3, from 2 independent experiments. **c)** Number of barcodes detected in the lungs, liver and CTCs of the MDA-MB-231 model for each mode of injection. **d)** Shannon diversity index of metastases in the MDA-MB-231 model. IMFP n=14, IMFPc n=10, SC n=8, ID n=9, IV n=8, from 2 to 3 independent experiments. **e)** Hierarchical clustering of Pearson correlation values for MDA-MB-231 lung metastases from all experiments (top panel) or for each experiment (bottom panels). **f)** Number of barcodes and **g)** Shannon diversity index of primary tumours and lung metastases in the MDA-MB-231 model, depending on the mode of injection. **b-g**) One-way ANOVA followed by Tukey multiple comparison test, ns (non significant)=p value>0.05, *=p value <0.05, **=p value <0.005, ***=p value <0.0005, ****=p value <0.0001. Error bars represent the standard deviation of the means (SD) and the shape represents independent experiments.

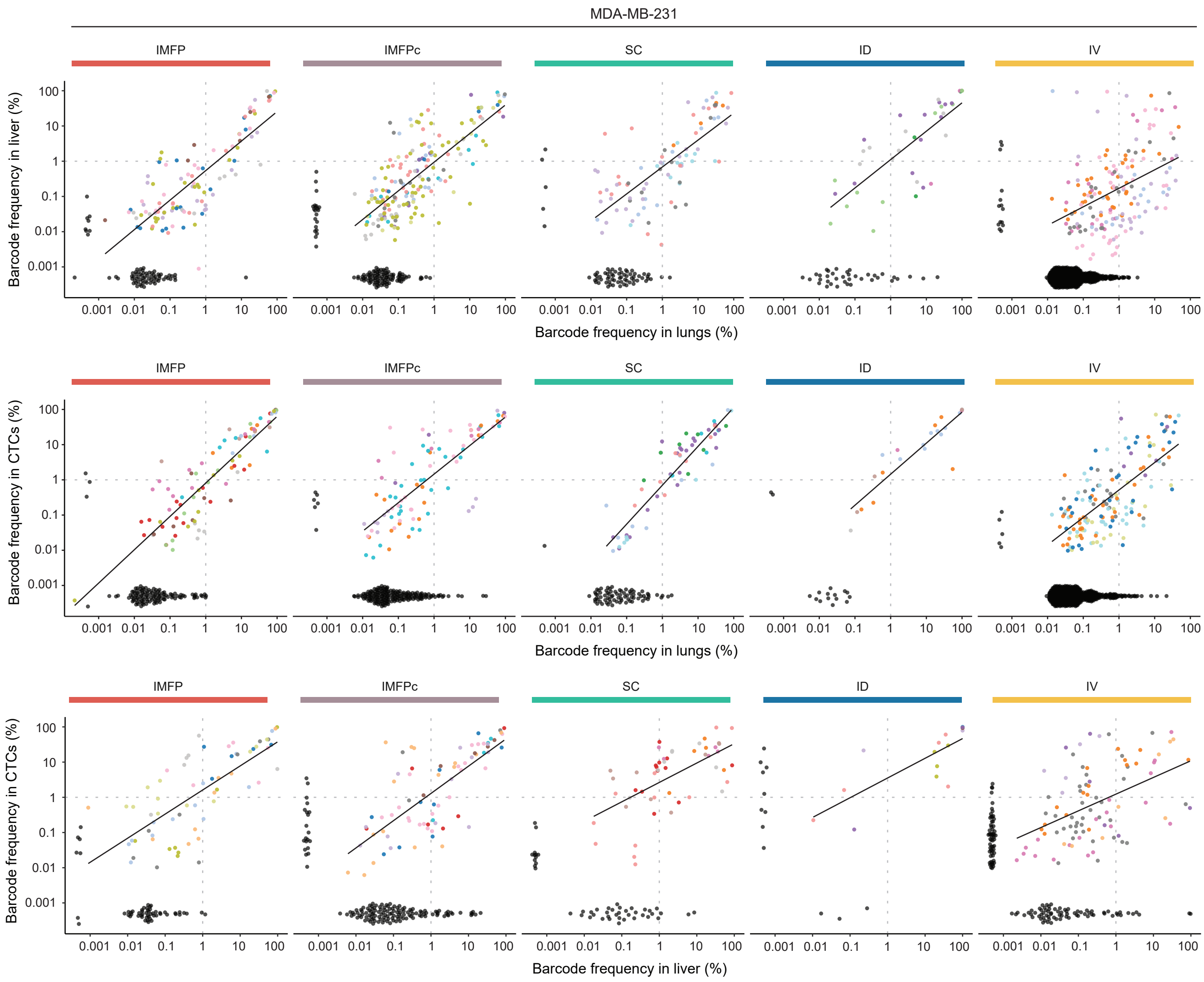

Supplementary Figure 7. Relationship between barcode frequency in lungs and liver (top panel), lungs and CTCs (middle panel), and liver and CTCs (bottom panel) for MDA-MB-231 model. The barcode repertoire is represented in the scatter plots (each dot is a barcode) for each mode of injection. Each colour represents an individual mouse. Black dots on the x-axis and y-axis represent barcodes uniquely found in these organs. Dashed lines indicate a frequency of 1% in organs.

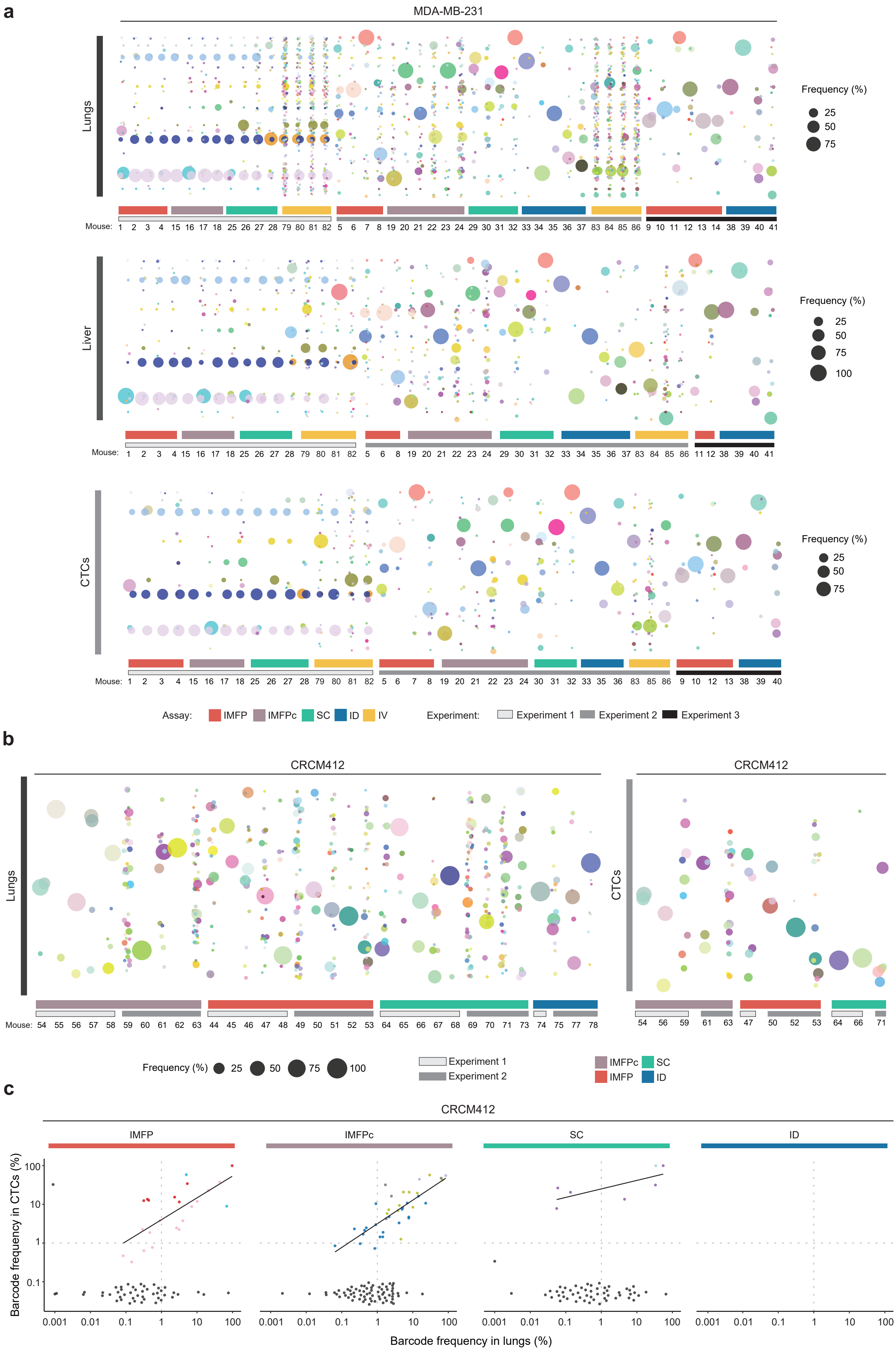

Supplementary Figure 8. a) Bubble plots representing the individual barcode repertoire detected in each metastatic site for each mode of injection in the MDA-MB-231 model. Each dot is a barcode, and the size of the dot is proportional to the frequency of the barcode in the indicated organ (scale on the right). Barcodes are randomly distributed on the y-axis. The localisation and colour of a given barcode are conserved across the bubble plots. **b)** Individual barcode composition represented as bubble plots for each distant site and mode of injection for the CRCM412 model. **c)** Relationship between barcode frequency in lungs and CTCs in the PDX CRCM412 model for each mode of injection. Each colour represents an individual mouse.
