# Supplementary material for: Experimental and spontaneous metastasis assays can result in divergence in clonal architecture
Source: Commun Biol. 2023 Aug 7;6:821. doi: 10.1038/s42003-023-05167-5 (PMC10406815; doi:10.1038/s42003-023-05167-5)
Supplement: Supplementary file 2 — Description of Additional Supplementary Files [file 42003_2023_5167_MOESM2_ESM.pdf]

## Description of Additional Supplementary Files

**File name:** Supplementary Data 1
**Description:** Primer sequences for second barcode PCR

**File name:** Supplementary Data 2
**Description:** The source data behind the graphs in the paper
